# Resveratrol Protects against Sepsis-Associated Encephalopathy and Inhibits the NLRP3/IL-1*β* Axis in Microglia

**DOI:** 10.1155/2016/1045657

**Published:** 2016-01-26

**Authors:** Da-ming Sui, Qun Xie, Wen-jing Yi, Sahil Gupta, Xi-ya Yu, Jin-bao Li, Jun Wang, Jia-feng Wang, Xiao-ming Deng

**Affiliations:** ^1^Department of Anesthesiology and Intensive Care, Changhai Hospital, Second Military Medical University, 168 Changhai Road, Shanghai 200433, China; ^2^Department of Anesthesiology, Chengdu Military General Hospital, 270 Tianhui Road, Chengdu 610083, China; ^3^The Keenan Research Centre for Biomedical Science, Li Ka Shing Knowledge Institute, St. Michael's Hospital, 209 Victoria Street, Toronto, ON, Canada M5B 1T8; ^4^Department of Surgery, St. Michael's Hospital, University of Toronto, 30 Bond Street, Toronto, ON, Canada M5B 1W8

## Abstract

Sepsis-associated encephalopathy (SAE) is characterized as brain dysfunction associated with sepsis. In this study we sought to investigate the effects of resveratrol in mice with SAE, as well as its effects in NLRP3 inflammasome and IL-1*β*, which were critical in the pathogenesis of SAE. SAE was induced in mice via cecal ligation and puncture (CLP), and resveratrol was administered at two doses after surgery. Spatial learning memory functions were evaluated by Morris water maze testing. Apoptosis in the hippocampus was quantified using TUNEL assay. Inflammation in the hippocampus was quantified by measuring the levels of microglial activation, NLRP3, and IL-1*β*. CLP mice treated with resveratrol demonstrated a better spatial memory during water maze training. The TUNEL assay demonstrated significantly attenuated rates of apoptosis, in resveratrol treated mice, while decreasing the number of iba-1 positive microglia in the hippocampus region. NLRP3 expression and IL-1*β* cleavage were well inhibited by resveratrol dose-dependently. The* in vitro* results showed that in the BV2 cell lines resveratrol prevents ATP induced NLRP3 activation and IL-1*β* cleavage, which were reversed by the sirtuin 1 inhibitor, nicotinamide. In conclusion, resveratrol improves the spatial memory in mice with SAE and inhibits the NLRP3/IL-1*β* axis in the microglia.

## 1. Introduction

Sepsis refers to the systemic inflammatory response by the immune system towards invading foreign pathogens. This multipronged defense response is the third leading cause for death in the United States [1]. During this response, multiple organ failure has the potential to be elicited, where manifestation of brain dysfunction during sepsis can lead to sepsis-associated encephalopathy (SAE) [2, 3]. SAE is defined as diffuse cerebral dysfunction during sepsis, without explicit evidence of microbial infection within the central nervous system, intracranial defects, or encephalopathy induced by hepatic or renal factors [3]. The incidence of SAE is reported to be as high as 70% and the clinical symptoms may vary from cognitive dysfunction and delirium to deep coma. The presence of SAE is seen to occur in parallel with extensive treatment time in the intensive care unit (ICU) and a higher mortality rate [4]. Despite the overwhelming amounts of deaths associated with SAE, an effective therapy against SAE is still lacking. Majority of the treatments directed towards SAE patients are focused towards controlling the systemic spread of infection, while providing supportive therapy.

The etiologies contributing towards SAE, as concluded by Gofton and Young [3], include inflammatory cytokines, microscopic brain injury, blood-brain barrier (BBB) compromise, altered cerebral metabolism, and neurotransmission and cerebral microcirculation. Microglia are heavily activated during SAE, which are dependent on neural, humoral pathways or BBB damage. At this time, patients also experience an increase in the secretion of cytokines, nitric oxide, and reactive oxygen species (ROS) in the brain parenchyma [3, 5]. IL-1*β* is a vital cytokine that mediates brain dysfunction by activating type 1 IL-1 receptor, ultimately preventing long-term potentiation deficiency in the hippocampus of septic mice [6–8].

Resveratrol is a natural phenol that is extracted from the skin of grapes, which are commonly used in red wine production, where they are advertised to have antiaging benefits for consumers [9, 10]. The biological role of resveratrol is to initiate the activation of sirtuin 1 (Sirt1), which is a class of deacetylase that epigenetically modifies and inactivates the acetylation of inflammatory proteins. Though the role of resveratrol in sepsis prognosis remains to be controversial, some studies suggest that resveratrol might be able to improve renal and myocardial functions, in murine model of sepsis [11, 12]. Moreover, resveratrol was also reported to be a potential therapy for neurodegenerative diseases through the inhibition of microglia activation [13, 14]. Therefore, our study attempted to investigate the role of resveratrol in development of SAE and NLRP3/IL-1*β* axis in microglia.

## 2. Methods

### 2.1. Animals

Male C57BL/6 mice, aged 6–8 weeks, were provided by the Experimental Animal Center at the Second Military Medical University. All experimental animal studies were approved by the local Animal Care and Use Committee of the Second Military Medical University. Sepsis was induced by the cecal ligation and puncture (CLP), as per procedures previously described by our laboratory [15, 16]. In general, all mice were subjected to sevoflurane anesthesia, in supine position, after a week worth of acclimatization to the lab environment. After skin sterilization, the skin and abdominal membrane were opened using scissors to expose the cecum, which was further ligated at half the distance to the end with a 1-0 Prolene thread. The ligated cecum was then punctured once with a 20 G needle and intestinal content was pushed out of the cecum. Then, the abdominal membrane and skin were closed in two layers. In the Sham-operated mice, the cecum was exposed in a similar fashion to the CLP procedure, without ligation and puncture. After surgery, all mice were provided with free access to water and food after recovery from anesthesia. In the animal experiments, mice were randomly allocated to one of four groups: Sham group, CLP group, Res 30 mg/kg group, and Res 10 mg/kg group (*n* = 6 for each group). In both Res groups, the mice were treated with four abdominal infusions of 30 mg/kg and 10 mg/kg resveratrol (Winherb Medical Tech., Shanghai, China) in dimethyl sulfoxide (DMSO) (Sigma-Aldrich, St. Louis, USA) saline solution at 1 hour prior to surgery and again at 6 h, 12 h, and 18 h after surgery. The Sham and CLP groups were treated with four times of DMSO infusions at the same concentration.

### 2.2. Cell Culture

The mouse BV2 cell lines were purchased from the Tongpai Biological Technology (Shanghai, China) as an alternative to investigate microglia* in vitro*. BV2 cells, at passages 10–15, were cultured in Dulbecco's modified eagle medium/F-12 (DMEM/F-12, Gibco, Langley, USA) with 10% fetal bovine serum (Sigma-Aldrich, St. Louis, USA), 1% penicillin, and streptomycin (Gibco, Langley, USA). In the first set of* in vitro* experiments, the cell populations were allocated into one of four groups: control group, lipopolysaccharide (LPS) group, Res1 group, and Res2 group. Cells in the control group were treated with DMSO + adenosine triphosphate (ATP) (100 *μ*M, Sigma-Aldrich, St. Louis, USA), DMSO + LPS (100 ng/mL, Sigma-Aldrich, St. Louis, USA) + ATP (100 *μ*M) in the LPS groups, resveratrol (30 *μ*M) + ATP (100 *μ*M) + LPS (100 ng/mL) in the Res 30 *μ*M group, and resveratrol (15 *μ*M) + ATP (100 *μ*M) + LPS (100 ng/mL) in the Res 15 *μ*M group. In the second set of* in vitro* experiments, the cell populations were allocated into one of four groups: control group, LPS group, Res group, and NAM group. The cells in the control group were treated with DMSO + ATP (100 *μ*M), DMSO + LPS (100 ng/mL) + ATP (100 *μ*M) in the LPS groups, resveratrol (30 *μ*M) + LPS (100 ng/mL) + ATP (100 *μ*M) in the Res group, and nicotinamide (10 mM, Sigma-Aldrich, St. Louis, USA) + resveratrol (30 *μ*M) + LPS (100 ng/mL) + ATP (100 *μ*M) in the NAM group.

### 2.3. Morris Water Maze Task

Morris water maze task was performed in a circular pool with a diameter of 100 cm and a height of 50 cm, in an isolated environment (Jiliang Software, Shanghai, China). Different shapes were marked on the inner walls of the pool to recognize the relative position of the mouse. Water (21.5 ± 0.5°C) containing food-grade titanium dioxide (Jianghu Taibai, Shanghai, China) was filled into the pool to a height of the three quarters of the wall. The pool was also divided into four quadrants and monitored with a video camera on the top. A platform with a diameter of 7 cm was place into one of the four quadrants, 1 cm below the water surface.

CLP or Sham-operated mice were trained for Morris water maze task from the 4th day to 7th day after surgery. The mice were place on the platform for a total of 10 seconds and were then removed from the pool. In the subsequent training session, the mice were individually placed into each of the 4 quadrants and were allowed to search the platform for a period of 60 seconds. If the mice were unable to reach the platform within 60 seconds, they would be placed on the platform for an additional 10 seconds. The escape latency, distance of swimming, and time spent in the target quadrant were recorded for each training process.

Half an hour after the last training session on the 7th day, the mice were subjected to the probe trial with the platform removed from the pool. All mice were monitored for 60 seconds in order to observe the distance of swimming, time spent in the target quadrant, and frequency of crossing the platform.

### 2.4. Terminal Deoxynucleotidyl Transferase dUTP Nick End Labeling (TUNEL)

TUNEL assay was performed to determine the apoptotic rates of the cell, in the hippocampus region of the mouse using the In-Site Cell Death Detection Kit (Roche, Basel, Switzerland). In general, the hippocampus region was isolated from the brain tissue at 24 hours after surgery and was fixed using 4% paraformaldehyde for 24 hours, allowing sections to be prepared. The sections were deparaffinized with xylene, hydrated with ethanol, and pretreated with proteinase K. The endogenous peroxidase was blocked with 3% H_2_O_2_, after which sections were incubated in terminal deoxynucleotidyl transferase (TdT) reaction mixture. In the negative control group, TBS solution was used instead of TdT. The sections were then incubated with streptavidin-HRP, colorized diaminobenzidine (DAB), and counterstained with bis-benzamide. The apoptotic rate was represented by the average number of TUNEL-positive cells in each field.

### 2.5. Immunofluorescence (IF) Assay

Paraffin sections of the hippocampus regions were prepared for the IF assay. After deparaffinization and rehydration, the sections were incubated in citrate buffer to retrieve antigen by heat-mediated method. The sections were then blocked with 3% bovine serum albumin (Sigma-Aldrich, St. Louis, USA) for 30 minutes and incubated in the primary antibody diluted at 1 : 100 (rabbit anti-mouse anti-iba-1 antibody, Abcam, Cambridge, UK) at 4°C overnight and lastly in Cy3 labeled secondary antibody diluted at 1 : 400 at room temperature for 60 minutes. Then the slides were incubated in the primary antibody against iba-1 antibody (1 : 100, Abcam, Cambridge, UK) at 4°C overnight and in Alex488 labeled secondary antibody diluted at 1 : 400. The nucleus was stained with Hoechst (Beyotime, Nantong, China). Finally, the slides were dehydrated and mounted for detection under the inverted fluorescence microscope (Leica, Wetzlar, Germany).

### 2.6. Reverse Transcription Polymerase Chain Reaction (RT-PCR)

Hippocampus tissue harvested at 24 hours after surgery was homogenized in 500 *μ*L TRIZol (Invitrogen, Carlsbad, USA) and the total RNA were purified by chloroform/isopropanol method. The RNA concentration was determined by nanodrop and adjusted to 0.5 *μ*g/*μ*L. Reverse transcriptase was performed using the PrimeScript RT reagent Kit (Takara, Dalian, China) and cDNA was stored at −80°C. The quantitative real-time PCR was performed using the SYBR Advantage PCR Premix (Takara, Dalian, China) in a 20 *μ*L system. The results were normalized to GAPDH and were expressed as the value of 2^−ΔΔCt^. The primers for NLRP3, IL-1*β*, and GAPDH were as follows:

  NLRP3: Forward 5′-ATGCTGCTTCGACATCTCCT-3′, Reverse 5′-GTTTCTGGAGGTTGCAGAGC-3′;
  IL-1*β*: Forward 5′-GCCCATCCTCTGTGACTCAT-3′, Reverse 5′-AGGCCACAGGTATTTTGTCG-3′;
  GAPDH: Forward 5′-ACCCAGAAGACTGTGGATGG-3′, Reverse 5′-CACATTGGGGGTAGGAACAC-3′.

### 2.7. Western Blot Assay

The hippocampus tissue was homogenized while the cells were lysed in lysis buffer with protease inhibitor (Sigma-Aldrich, St. Louis, USA). 30 *μ*g of protein was loaded for each sample, which was then subjected to 10% SDS-PAGE electrophoresis. The gel was then transferred to a polyvinylidene fluoride membrane (BD Bioscience, Franklin Lakes, USA). After blocking with 5% skimmed milk, the membrane was incubated on a rocker with primary antibody at 4°C overnight. Secondary antibody was added the following day at room temperature for 2 hours. Membranes were then treated with ECL reagent (ThermoFisher, Waltham, USA) to detect protein expression, using the UVP BioImaging system (Upland, USA). The densitometry was analyzed using the Image-Pro Plus software (Media Cybernetics, Bethesda, USA). The antibodies for Western blot included the rabbit anti-mouse anti-IL-1*β* antibody (1 : 1000, Abcam, Cambridge, UK), rabbit anti-mouse anti-iba-1 antibody (1 : 1000, Abcam, Cambridge, UK), rat anti-mouse NLRP3 antibody (1 : 1000, R&D, Minneapolis, USA), rabbit anti-mouse *β*-actin (1 : 1000, Abcam, Cambridge, UK), HRP-conjugated goat anti-rabbit IgG antibody (1 : 4000, Cell Signaling Technology, Danvers, USA), and HRP-conjugated goat anti-rat IgG antibody (1 : 4000, R&D, Minneapolis, USA).

### 2.8. Statistical Analysis

All statistical analyses were performed using the GraphPad Prism 5.0 software (La Jolla, USA). All data are represented as mean ± standard derivation (SD). The latency, distance, and time during the water maze training were compared using the two-way ANOVA test with Bonferroni's correction. The other comparisons among four groups were performed by the one-way ANOVA test, followed by LSD post hoc analyses. *P* < 0.05 was regarded as statistically significant.

## 3. Results

### 3.1. Resveratrol Improves the Spatial Learning and Memory of Septic Mice

**Figure 1:**
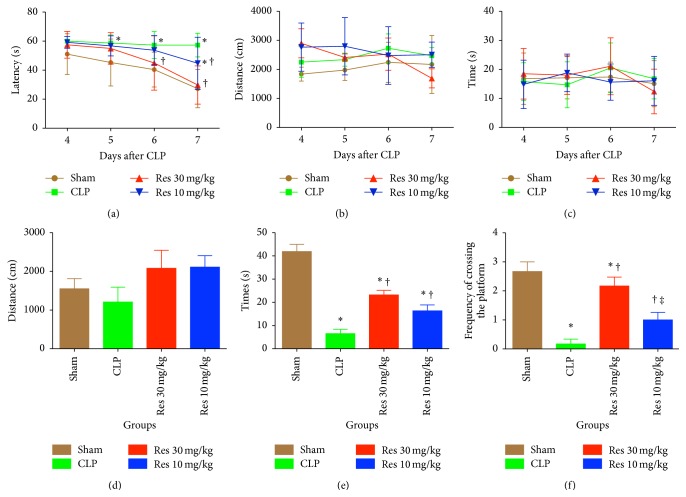
Morris water maze training (a)–(c) and hidden platform tests (d)–(f). (a) Latency to escape; (b) total distance of swimming; (c) time spent in the target quadrant; (d) distance of swimming; (e) times spent in the target quadrant; (f) frequencies of platform crossing. Res1 was treated with a higher dose of resveratrol (30 mg/kg), while Res2 was treated with a lower dose of resveratrol (10 mg/kg). ^*∗*^ *P* < 0.05 compared with Sham group, ^†^ *P* < 0.05 compared with CLP group, ^‡^ *P* < 0.05 compared with Res1 group, by two-way ANOVA test (a)–(c) or one-way ANOVA and LSD test (d)–(f). Bars represent the mean ± SD.

Morris water maze training was performed for a total of 4 days, after CLP or Sham surgery. In comparison to the Sham mice, CLP mice demonstrated significantly prolonged escape of latency from the fifth day after surgery (Figure 1(a)). CLP mice treated with a high dose (30 mg/kg) of resveratrol had shorter latency periods at days 3 and 4 of training (*P* < 0.05), while those treated with a lower dose of resveratrol (10 mg/kg) exhibited shorter latency periods on the last day of training (*P* < 0.05). It should be noted that the distance and time spent in the target quadrant were similar over the four days of training across all four groups (Figures 1(b) and 1(c)).

After the water maze training, the platform was removed to test the distance, time spent in the target quadrant, and frequency of crossing. The distance travelled was similar among the four groups (*P* > 0.05) (Figure 1(d)). CLP mice spent the shortest amount of time in the target quadrant (6.47 ± 4.63 s), which was significantly less than that of the Sham-operated (41.89 ± 7.53 s) (*P* < 0.05) and resveratrol treated CLP mice (Res1: 23.17 ± 4.96 s; Res2: 16.35 ± 6.19 s) (*P* < 0.05 for both). Also, the two doses of resveratrol did not affect the time spent in the target quadrant (*P* > 0.05). The time spent in the target quadrant in both resveratrol groups was shorter than in Sham group, while it was longer than in CLP group (*P* < 0.05) (Figure 1(e)). The frequency of crossing the platform was reduced in CLP mice (0.17 ± 0.41) in comparison to the Sham-operated mice (2.67 ± 0.82) (*P* < 0.05), while resveratrol significantly increased the frequency, in a dose response manner (Res1: 2.17 ± 0.75; Res2: 1.00 ± 0.63) (Res1 versus CLP, *P* < 0.05; Res2 versus CLP, *P* < 0.05; Res1 versus Res2, *P* < 0.05) (Figure 1(f)).

### 3.2. Resveratrol Alleviates Apoptosis in the Hippocampus Region of Septic Mice

**Figure 2:**
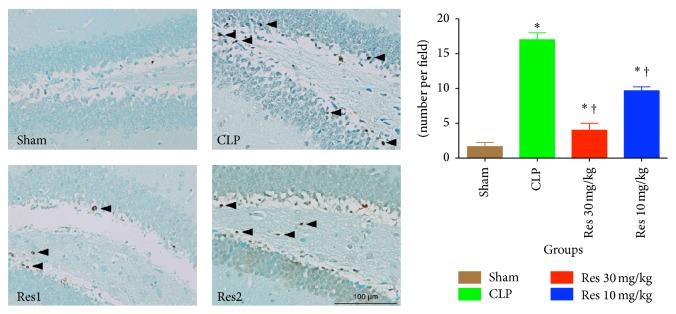
Apoptosis in the hippocampus of septic mice by the TUNEL assay. Resveratrol was seen to significantly decrease the rate of apoptosis of cells in the hippocampus, in relation to the CLP treated group. ^*∗*^ *P* < 0.05 compared with Sham group and ^†^ *P* < 0.05 compared with CLP group by one-way ANOVA and LSD tests. Bars represent the mean ± SD.

Pathological changes in septic mice were determined by measuring the rates of apoptosis and microglial activation in the hippocampus. TUNEL assay showed that the apoptotic rates of cells were significantly increased during sepsis, 17.0 ± 1.0 versus 1.67 ± 0.58 per field (*P* < 0.05). Resveratrol was seen to effectively inhibit significant increases in apoptosis rates, 4.0 ± 1.0% in Res 30 mg/kg group and 9.7 ± 0.6% in Res 10 mg/kg group (*P* < 0.05 for comparisons between Res1 or Res2 group versus CLP or Sham group) (Figure 2).

### 3.3. Microglial Activation in the Hippocampus Region Is Inhibited by Resveratrol

**Figure 3:**
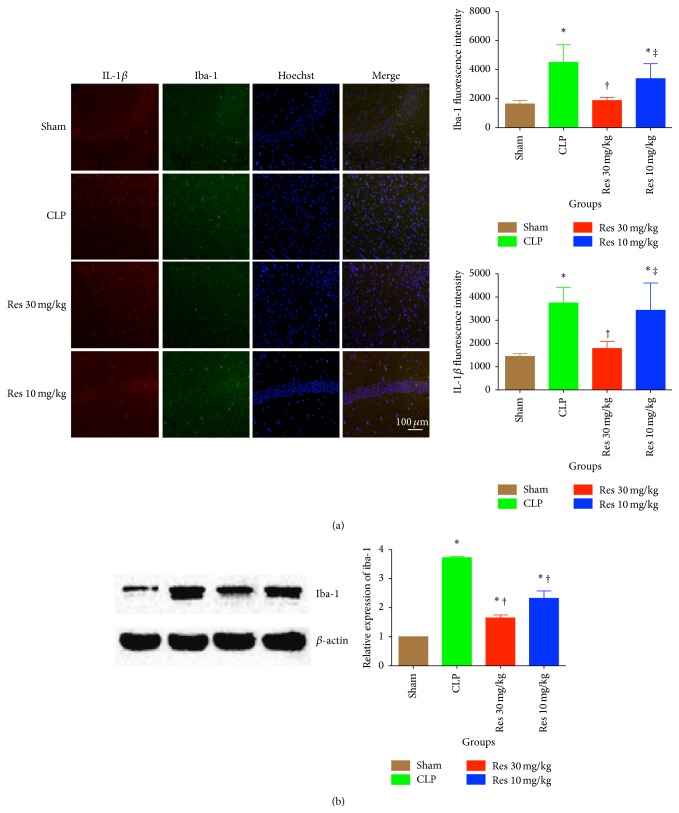
Microglia activation is increased in the hippocampus during SAE but it attenuated in the presence of resveratrol. Resveratrol is able to appropriately modulate the expression and activation of microglia in the hippocampus of CLP treated mice. ^*∗*^ *P* < 0.05 compared with Sham group, ^†^ *P* < 0.05 compared with CLP group, and ^‡^ *P* < 0.05 compared with Res1 group, by one-way ANOVA and LSD tests. Bars represent the mean ± SD.

Iba-1 is a marker of activation for microglia [17]. Relative expression of iba-1 in the hippocampus region was detected by both IF and Western blot assay. Iba-1 fluorescence intensity as shown by IF assay was significantly increased in CLP mice, while 30 mg/kg resveratrol reduced the intensity significantly (*P* < 0.05). IL-1*β* was also inhibited significantly by 30 mg/kg resveratrol (*P* < 0.05) (Figure 3(a)). The protein expression of iba-1 was further confirmed by Western blot. Resveratrol reduced the expression of iba-1 at both doses, but there were no significant changes between the two Res groups (*P* < 0.05 for all comparisons between Sham group and other groups, *P* < 0.05 for comparisons between CLP group and both Res groups, *P* > 0.05 between Res1 and Res2 groups) (Figure 3(b)).

### 3.4. Resveratrol Prevents NLRP3 Expression and IL-1*β* Cleavage in the Hippocampus of Septic Mice

**Figure 4:**
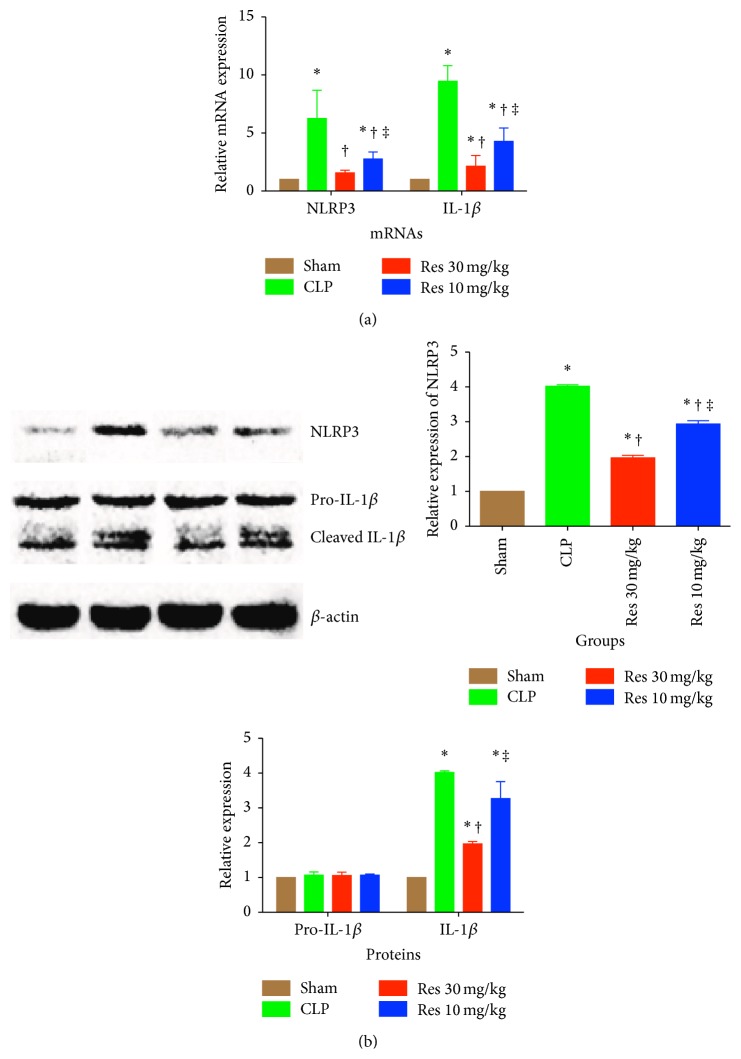
IL-1*β* and NLRP3 inflammasome are upregulated during SAE. (a) mRNA assay of NLRP3 and IL-1*β* by quantitative real-time PCR; (b) Western blot assay of NLRP3 and IL-1*β*, which has been normalized to *β*-actin. ^*∗*^ *P* < 0.05 compared with Sham group, ^†^ *P* < 0.05 compared with CLP group, and ^‡^ *P* < 0.05 compared with Res1 group, by one-way ANOVA and LSD tests. Bars represent the mean ± SD.

The NLRP inflammasome is a type of NOD-like receptor (NLR), which is known to activate caspase-1, which promotes the cleavage of IL-1*β* [18]. We detected the expression of both NLRP3 and IL-1*β* at both mRNA and protein levels in the hippocampus. At the mRNA level, resveratrol reduced the NLRP3 and IL-1*β* expression in a dose dependent manner, while the NLRP3 level in Res 30 mg/kg group was reduced to a similar level to that in Sham group (Figure 4(a)). NLRP3 protein levels were reflective and followed the expression levels found at the mRNA level. Resveratrol was able to significantly reduce the expression of NLRP3, in a dose dependent manner. Interestingly, the IL-1*β* levels were also significantly reduced in Res1 group at the level of cleaved protein but not at the pro-form level (Figure 4(b)).

### 3.5. Resveratrol Inhibits IL-1*β* by Activating Sirt1 in BV2 Cell Lines

**Figure 5:**
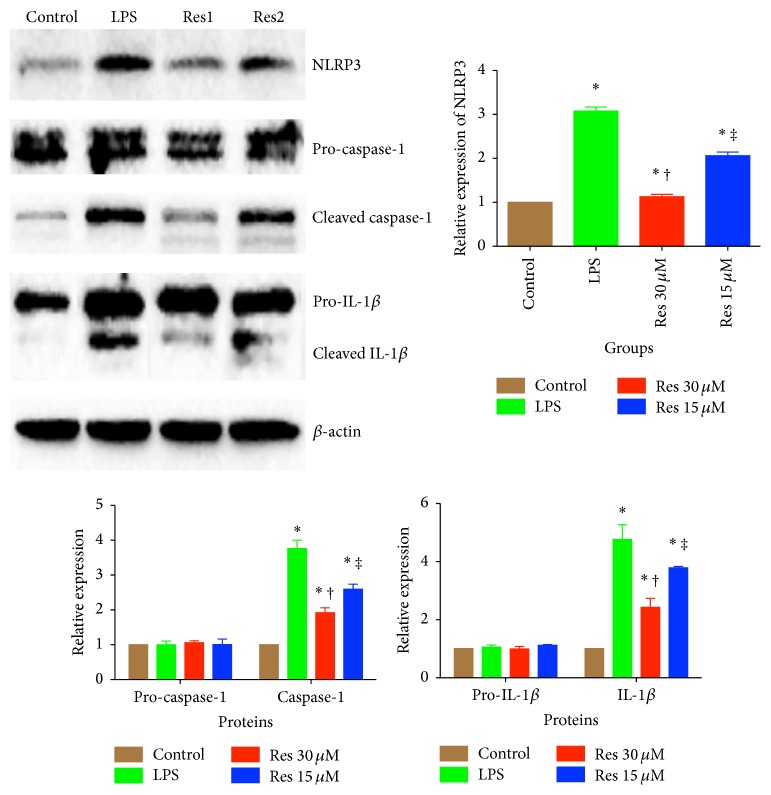
*In vitro* assay of the inhibitory effect of resveratrol on inflammasome activation in BV2 cells. BV2 immunoblots of NLRP3, caspase-1 and IL-1*β*, in the presence and absence of resveratrol. All blots have been normalized to *β*-actin. Control cells were stimulated with ATP (100 *μ*M) alone, while LPS cells were stimulated with both ATP and LPS (100 ng/mL). Res1 cells were treated with higher dose of resveratrol (30 *μ*M), in the presence of ATP and LPS. Res2 cells were treated with lower dose of resveratrol (15 *μ*M), in the presence of both ATP and LPS. ^*∗*^ *P* < 0.05 compared with control group, ^†^ *P* < 0.05 compared with LPS group, and ^‡^ *P* < 0.05 compared with Res1 group, by one-way ANOVA and LSD tests. Bars represent the mean ± SD.

**Figure 6:**
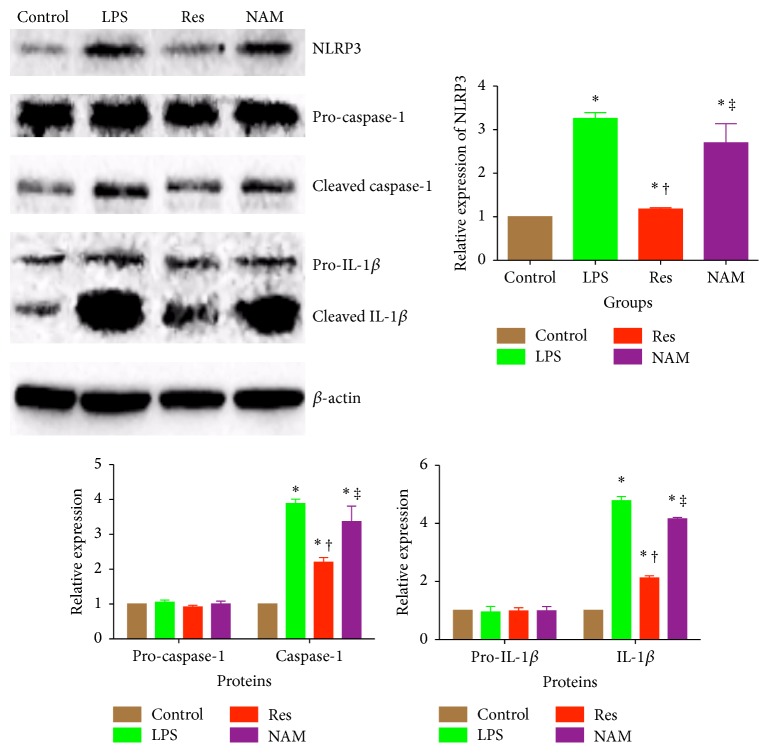
*In vitro* assay of the effects of Sirt1 inhibitor, NAM, on the protective effects of resveratrol in BV2 cells. Blots portray protein expression of NLRP3, caspase-1, and IL-1*β*. All immunoblots were normalized to the loading control, *β*-actin. This population of BV2 cells was treated with resveratrol, either in the presence or in the absence of nicotinamide. Control cells were stimulated with ATP alone, while LPS cells were stimulated with both ATP and LPS. Res cells were treated with one dose of resveratrol at a concentration of 30 *μ*M, in the presence of both ATP and LPS. NAM cells were treated with the same dose of resveratrol (30 *μ*M) but in the presence of nicotinamide (10 mM), ATP, and LPS. ^*∗*^ *P* < 0.05 compared with control group, ^†^ *P* < 0.05 compared with LPS group, and ^‡^ *P* < 0.05 compared with Res group, by one-way ANOVA and LSD tests. Bars represent the mean ± SD.

*In vitro* study of microglia was performed using the BV2 cell lines. BV2 cells were stimulated with ATP to prime the NLRP3 (±LPS) and two doses of resveratrol. In the presence of LPS and ATP, the NLRP3 expression significantly upregulated and the cleavage of caspase-1 and IL-1*β* were enhanced; however, in the presence of resveratrol, we were able to significantly inhibit such processes (Figure 5). In order to investigate if the protective role of resveratrol was dependent on Sirt1, BV2 cells were blocked with the Sirt1 inhibitor, NAM. The usage of the NAM inhibitor completely abolished the inhibition of NLRP3 expression and IL-1*β* cleavage, which was induced by resveratrol (Figure 6).

## 4. Discussion

In this study we have demonstrated that resveratrol improves the spatial learning and memory capacity of septic mice. These conclusions were made based on the attenuation of neuronal apoptosis and microglial activation, after resveratrol administration. Moreover, resveratrol was demonstrated to inhibit the NLRP3/IL-1*β* axis at both* in vivo* and* in vitro* levels.

Recent literature reports indicate that resveratrol has been considered for optimal usage for neuroprotective effects [10]. It was shown that resveratrol preconditioning reduced the infarction volume, while improving the neurological score in the mouse after middle cerebral artery occlusion, thereby providing an extended ischemia tolerance in the brain [19, 20]. Resveratrol was also reported to protect against poststroke depression through the regulation of the hypothalamus-pituitary-adrenal axis in rats [21]. Additional studies regarding the neuroprotective effect were focused on exploring the antiaging and antineurodegerative effect [14, 22]. Resveratrol was demonstrated to reduce p53 expression, nuclear factor-*κ*B activation, and tau phosphorylation and improve cognitive function in the senescence-accelerated prone mouse, a model with accelerated aging and cognitive decline [23]. In another common neurodegenerative disease, Parkinson's disease, resveratrol was able to increase the motor coordination skills in rat models [24]. But most importantly, in humans, a controlled randomized, pair-matched, placebo-controlled trial showed that continuous intake of resveratrol for a period of 26 weeks showed improvements in memory performance, which included memory retention, delayed recall, recognition, learning ability, and fifth learning trial [25]. The above-mentioned studies provide strong evidence for resveratrol's ability to provide protection against neuronal impairment caused by stroke, neurodegenerative factor, and aging. Therefore, not surprisingly, our data demonstrated that resveratrol was protective against SAE according to the spatial learning training and test, as well as the neuronal apoptosis in the hippocampus.

Microglia activation in SAE has been well documented in an autopsy study, which showed a significantly increase in the expression of CD68 in both the cortex and white matter of patients dying from sepsis [5]. Michels et al. [26] demonstrated that minocycline, an inhibitor for microglia activation, reduced oxidative stress and inflammatory burden in the hippocampus, while improving the long-term cognitive behavior of CLP mice. Another study showed that IL-1*β* expression was increased by the microglia of aged mice that were challenged with peripheral LPS administration [27]. The IHC and Western blot assays in our present study showed that resveratrol administration reduced the expression of both iba-1 and IL-1*β*, which may potentially be a pivotal mechanism involved in the protective effect.

IL-1*β* secretion is controlled by the activation of NLRP3 inflammasomes, which recognize the pathogen-associated molecular pattern (PAMP) or danger-associated molecular pattern (DAMP), such as ROS. NLRP3 activation promotes the cleavage of procaspase-1, in the presence of the adaptor protein called ASC. Mature caspase-1 then promotes the proteolytic cleavage of pro-IL-1*β* at Asp116 into IL-1*β*. NLRP3/caspase-1 axis played a central role in the pathogenesis of Alzheimer's disease [28]. The accumulation of amyloid-*β* can activate NLRP3 in microglia, while the increased maturation of IL-1*β* further hampered the clearance of amyloid-*β* by microglia [29]. Our data suggest that NLRP3/IL-1*β* might also participate the development of SAE and that resveratrol ameliorates SAE through the reverse activation of NLRP3. The role of resveratrol on NLRP3 activation has also been demonstrated in macrophages and microglial cells in different cell or disease models [30, 31].

We believe that there are several mechanisms involved in the reduction of NLRP3 expression and IL-1*β* maturation by resveratrol. Sirt1 was reported to deacetylate nuclear factor-*κ*B, thereby inhibiting the translocation of the p65 subunit to the nucleus [32, 33]. Therefore, our findings can be considered parallel to those in literature as resveratrol was observed to decrease the expression of NLRP3 and IL-1*β* mRNA. Sirt1 is a protein that assists in the clearance of ROS, which may potentially downregulate the activation of NLRP3 [34]. Therefore, we can potentially correlate the decreased activation of NLRP3 with a reduction in ROS, which was dependent on resveratrol administration. Furthermore, Misawa et al. [35] found that resveratrol might block the assembly of NLRP3 on endoplasmic reticulum and ASC on mitochondria, thereby inhibiting the activation of NLRP3 inflammasome.

There are several limitations in our present studies. Firstly, the exact role of NLRP3/IL-1*β* axis in the protective effects of resveratrol against SAE was not fully established. In order to determine the causal relation of NLRP3 inflammasome and protective role of resveratrol, NLRP3 knockout mice might be required to detect the effect of resveratrol on SAE in NLRP3 deficient mice. Secondly, which step of NLRP3 priming and activation did the resveratrol inhibit was not clarified. Resveratrol might inhibit NLRP3 activation by the above-mentioned mechanisms, but it may also inhibit the NLRP3 and IL-1*β* priming via sirt1, since sirt1 might prevent the transcriptional level of IL-1*β* [36]. Finally, NAM is an inhibitor for NAD dependent enzymes and sirt1 is one of the enzymes that can be inhibited by NAM. Thus the specificity issue may limit the conclusion that protective role of resveratrol is dependent on sirt1.

## 5. Conclusions

Our present study illuminates how resveratrol can be considered as a protective against SAE. Resveratrol can also inhibit the NLRP3/IL-1*β* axis in hippocampus of SAE mice and microglial cells stimulated by LPS and ATP, which is critical for the development of SAE. The exact mechanism underlying in the protective role of resveratrol against SAE remains to be further investigated.
